# Evaluation of Test to Stay Strategy on Secondary and Tertiary Transmission of SARS-CoV-2 in K–12 Schools — Lake County, Illinois, August 9–October 29, 2021

**DOI:** 10.15585/mmwr.mm705152e2

**Published:** 2021-12-31

**Authors:** Natsumi Nemoto, Soneet Dhillon, Steven Fink, Emily J. Holman, Amyanne Keswani Cope, Thu-Ha Dinh, Juliana Meadows, Dina Taryal, Funmilayo Akindileni, Megan Franck, Emily Gelber, Lea Bacci, Sana Ahmed, Ebony S. Thomas, John C. Neatherlin

**Affiliations:** ^1^CDC Foundation COVID-19 Corps, Waukegan, Illinois; ^2^Chicago Medical School, Rosalind Franklin University, North Chicago, Illinois; ^3^CDC COVID-19 Response Team; ^4^Lake County Health Department and Community Health Center, Waukegan, Illinois.

The COVID-19 pandemic has resulted in school closures and reduction of in-person learning (*1*). In August 2021, the Lake County Health Department (LCHD) in Illinois introduced a Test to Stay (TTS) strategy, whereby unvaccinated students, teachers, and staff members with certain school-related COVID-19 exposures could remain in school and participate in school-related extracurricular activities. Eligibility to participate in TTS required the following conditions to be met: 1) the exposure occurred while both the person with COVID-19 (index patient) and the close contact were masked; 2) the close contact remained asymptomatic, practiced consistent mask wearing, and maintained physical distancing; and 3) the close contact underwent testing for SARS-CoV-2 (the virus that causes COVID-19) on days 1, 3, 5, and 7 after exposure to the index patient. LCHD permitted kindergarten through grade 12 (K–12) schools in Lake County to implement TTS; 90 schools, representing 31 school districts in Lake County, implemented TTS during August 9–October 29, 2021. During the implementation period, 258 COVID-19 cases were reported. Among 1,035 students and staff members enrolled in TTS, the secondary attack risk (number of close contacts who received a positive SARS-CoV-2 test result within 14 days after exposure to an index patient, divided by total number of close contacts) was 1.5% (16 of 1,035). Among the 16 secondary cases identified, all were in students, and none appeared to transmit SARS-CoV-2 to other school-based contacts. However, nine tertiary cases were identified among household contacts of the 16 secondary cases, and four of the nine were fully vaccinated. Assuming a maximum of 8 missed school days for every 10-day quarantine period, up to 8,152 in-person learning days were saved among TTS participants. Implementation of TTS with other concurrent prevention strategies, including masking and physical distancing, limited further spread of SARS-CoV-2 within K–12 schools and allowed students to safely sustain in-person learning. Although vaccination remains the leading public health recommendation to protect against COVID-19 for those aged ≥5 years, schools might consider TTS as an option for allowing close contacts who are not fully vaccinated to remain in the classroom as an alternative to home quarantine.

In fall 2021, LCHD encouraged eligible schools in Lake County, Illinois, to implement TTS. School eligibility criteria included 1) ability to report test results, including index patients and school-based close contacts, to Illinois Department of Public Health (IDPH) and LCHD within 24 hours and 2) school staff members’ availability for interviews to provide details regarding school-related exposures. School-based close contacts of persons with COVID-19 were eligible to participate in TTS if both the person with COVID-19 (i.e., index patient) and the contact were masked during the exposure, and the exposure occurred at school or while participating in school-related extracurricular activities. Contacts who met eligibility criteria could participate in TTS if they remained asymptomatic, practiced consistent mask wearing, maintained physical distancing, obtained parental consent, and underwent SARS-CoV-2 testing at school or off campus* on days 1, 3, 5, and 7 after exposure. Asymptomatic TTS participants who received negative SARS-CoV-2 test results and adhered to TTS requirements, including mandatory masking, could ride the school bus and attend in-person learning and school-based extracurricular activities, including sports. TTS participants were required to quarantine at home for 14 days while not attending school or participating in school-based activities. Close contacts were defined as persons who were within 3 feet (0.9 meters) of a COVID-19 patient for ≥15 cumulative minutes over a 24-hour period.^†^ Close contacts who had unmasked exposures within 6 feet (1.8 meters) of a COVID-19 patient were not eligible for TTS and quarantined at home. Persons who were fully vaccinated or who received a COVID-19 diagnosis in the 90 days before exposure were not required to quarantine and were not eligible to participate in TTS.

Schools reported COVID-19 case and close contact information to LCHD via REDCap (version 11.2.6; Vanderbilt University). LCHD staff members called parents of close contacts to identify additional exposures outside of school. Data were supplemented with information from the Salesforce case investigation and contact tracing management system, Illinois’ National Electronic Disease Surveillance System (I-NEDSS), and the Illinois state vaccination registry. Among TTS participants, secondary cases were defined as contacts who received a positive SARS-CoV-2 test result by a contact within 14 days after exposure to an index patient. Secondary attack risk of TTS participants was defined as number of close contacts who received a positive SARS-CoV-2 test result within 14 days after exposure divided by total number of close contacts. Estimated in-person learning days saved from TTS was calculated assuming a maximum of 8 missed school days for every 10-day quarantine. All analyses were performed using SAS software (version 9.4; SAS Institute). This activity was reviewed by CDC and was conducted consistent with applicable federal law and CDC policy.^§^

During August 9–October 29, 2021, 90 Lake County schools implemented TTS, representing 53.7% (6,267) of staff members and 53.4% (65,384) of public school students in Lake County (*2*). During this period, 258 index COVID-19 patients and 1,664 close contacts were reported. Among 1,068 close contacts eligible for TTS, 1,035 (96.9%) participated (Figure). Among TTS participants, 16 secondary cases were identified, all of whom were in students (Table 1); no secondary cases were identified among staff members. Eleven of the 16 secondary cases occurred among males, and nearly all cases were in non-Hispanic White students. The overall secondary attack risk was 1.5% (16 of 1,035).

**FIGURE:**
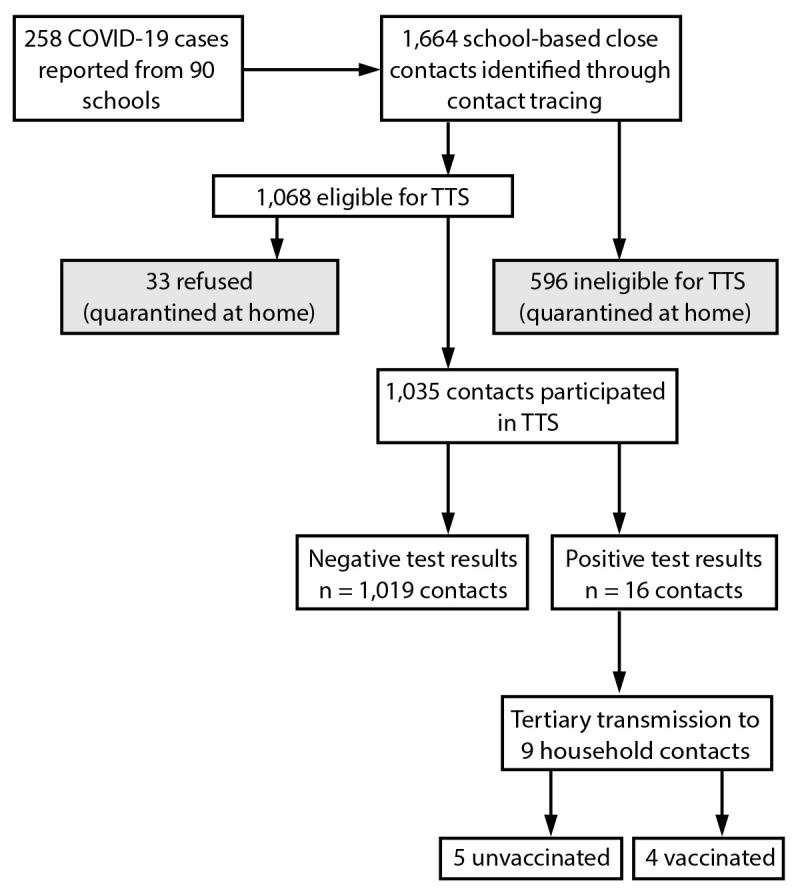
Identification of students and staff members who received a positive SARS-CoV-2 test result, school-based close contacts,* and SARS-CoV-2 test results among close contacts — Lake County, Illinois, August 9–October 29, 2021 **Abbreviation:** TTS = Test to Stay program. * Ineligibility for TTS includes unmasked exposures within 6 feet of a person with a case of COVID-19 and exposure to a person with a case of COVID-19 outside of school or school-related activities.

**TABLE 1:** Demographic characteristics of Test to Stay participants by SARS-CoV-2 test results — Lake County, Illinois, August 9–October 29, 2021

Characteristic	Test results of close contacts		
	Positive* (n = 16)	Negative (n = 1,019)	Total (N = 1,035)
**Student age, yrs, median (range)**	11 (5–16)	10 (3–18)	**10 (3–18)**
**Gender, no. (column %)**			
Female	5 (31.3)	467 (45.8)	**472 (45.6)**
Male	11 (68.8)	493 (48.4)	**504 (48.7)**
Unknown	0 (—)	59 (5.8)	**59 (5.7)**
**Race, no. (column %)**			
Black	2 (12.5)	35 (3.4)	**37 (3.6)**
White	14 (87.5)	662 (64.9)	**676 (65.3)**
Asian	0 (—)	71 (7.0)	**71 (6.9)**
American Indian or Alaska Native	0 (—)	7 (0.7)	**7 (0.7)**
Other	0 (—)	136 (13.4)	**136 (13.1)**
Unknown	0 (—)	108 (10.6)	**108 (10.4)**
**Ethnicity, no. (column %)**			
Hispanic/Latino	1 (6.3)	104 (10.2)	**105 (10.1)**
Not Hispanic/Latino	15 (93.8)	611 (60.0)	**626 (60.5)**
Other	0 (—)	117 (11.5)	**117 (11.3)**
Unknown	0 (—)	187 (18.3)	**187 (18.1)**

* This group represents secondary cases.

The 16 students with secondary cases received their positive test results on days 1 (three students), 2 (two), 3 (two), 4 (two), 5 (four), 6 (one), and 10 (two). Testing after day 7 occurred for those who missed the last day of TTS testing because of school holidays or weekends. Seven of the 16 students with secondary cases were symptomatic on the date of their positive test result, three developed symptoms after receiving a positive test result, and six remained asymptomatic. Based on investigation interviews, the most common likely locations^¶^ of COVID-19 exposure among TTS participants were school buses (56.3%), classrooms (32.4%), and school-sanctioned sports (7.4%); among these locations, the secondary attack risks were 1.5%, 0.6%, and 6.5%, respectively (Table 2). Secondary transmission was lowest in elementary schools (1.1%), followed by middle schools (1.3%) and high schools (4.9%).

**TABLE 2:** Grade level and exposure site characteristics of Test to Stay participants by SARS-CoV-2 test results — Lake County, Illinois, August 9–October 29, 2021

Characteristic	Test results of close contacts		
	Positive (n = 16)	Negative (n = 1,019)	Total (N = 1,035)
**Grade level/Staff members, no. (row %)**			
Teachers/Staff members	0 (—)	2 (100)	**2**
Elementary school students (grades K−5)	7 (1.1)	620 (98.9)	**627**
Middle school students (grades 6−8)	4 (1.3)	299 (98.7)	**303**
High school students (grades 9−12)	5 (4.9)	98 (95.1)	**103**
Location of exposure,* no. (row %)			
Classroom^†^	2 (0.6)	333 (99.4)	**335**
School bus	9 (1.5)	574 (98.5)	**583**
School-sanctioned sport	5 (6.5)	72 (93.5)	**77**
Extracurricular activity^§^	0 (—)	6 (100)	**6**
Unknown	0 (—)	34 (100)	**34**

* Exposure locations are mutually exclusive. School nurses reported one exposure site per close contact.

^†^ Classroom exposures include academic classes, indoor recess, physical education class, and staff meetings.

^§^ Extracurricular activities include drama club and band.

Assuming a maximum of 8 missed school days for every 10-day quarantine, TTS preserved up to 8,152 in-person learning days for TTS close contacts. None of the 16 secondary cases appeared to transmit SARS-CoV-2 to other school-based contacts. However, nine tertiary cases in five households were identified among household contacts of the 16 secondary cases; four of the nine were fully vaccinated (Figure).

## Discussion

Implementation of a TTS strategy with multiple prevention components, including masking and physical distancing, resulted in low secondary transmission of SARS-CoV-2 in K–12 schools in Lake County, Illinois. These findings highlight the usefulness of TTS to limit school-based transmission and sustain in-person learning (*1*,*3*,*4*). Previous research suggests that limited in-person instruction during the pandemic might have had a negative effect on learning and well-being among children (*5*).

Secondary transmission risk to students exposed during school-sanctioned sports was higher than that associated with classroom or school bus exposures. This is consistent with studies showing high transmission among sports participants (*6*,*7*). Also consistent with previous research (*8*), this study found that household contacts of persons exposed at school continue to be at risk for infection; among household contacts of the 16 secondary patients, nine tertiary cases were identified, four in fully vaccinated persons. Schools can help inform parents and guardians about the benefits of COVID-19 prevention strategies, including vaccination.

Although TTS can help limit in-school transmission of SARS-CoV-2, it is a resource-intensive strategy that might be difficult to implement because of increased administrative demands on staff members, requirements for robust contact identification and tracing, and testing availability. Low-resource schools might lack space for physical distancing during lunch, resulting in unmasked exposures within 6 feet, which would disqualify students from TTS eligibility, necessitating home quarantine. Among the 90 TTS schools in this study, 25.6% participated in a subsidized lunch program, compared with 38.1% of schools that did not implement TTS (*9*). Some schools reported a shortage of testing supplies, requiring TTS participants to access off-site testing, which might have presented a barrier in low-resource school settings. State and local public health and education agencies should strive to ensure that schools in low-resource areas have equitable access to staffing and testing supplies to implement TTS.

The findings in this report are subject to at least seven limitations. First, inequity in school districts’ staffing and testing resources might have introduced selection bias because only schools with sufficient resources offered TTS. High-resource schools might have more staffing capacity and physical spacing to apply prevention strategies (e.g., distancing students), which might have resulted in low transmission levels that are not generalizable to low-resource schools. Second, data might not be generalizable to areas with higher COVID-19 incidences and lower vaccination rates; COVID-19 incidence (7-day rolling average number of cases per 100,000 persons) in Lake County ranged from 59.7 to 217.1 over the evaluation period, with 53.5% of the total population vaccinated (*10*). Third, 33% of parents did not respond to LCHD calls or might have chosen not to disclose exposures occurring outside school, resulting in students at high risk being incorrectly enrolled in TTS. This would likely have resulted in an overestimation of secondary transmission among TTS participants. Fourth, testing days often deviated from testing cadence because school testing was not conducted on weekends. This deviation might have resulted in a delay of case and close contact identification. Fifth, teachers and staff members had much lower participation rates than did students in this evaluation because of high vaccination rates, low number of exposures meeting close contact definition, and lack of awareness in some schools that adults could participate in TTS. Sixth, households representing three secondary cases were unresponsive to attempted interviews to ascertain tertiary transmission, resulting in incomplete investigations. Finally, this analysis assumes that secondary attack risk represents direct transmission from contacts to cases. Given the unknown exposures and overlapping incubation and infectious periods that occur, distinct generations of transmission might be difficult to ascertain.

Implementation of TTS in coordination with other concurrent prevention strategies, including masking and physical distancing, allowed transmission of SARS-CoV-2 to remain low among K–12 schools in Lake County, Illinois, and saved up to 8,152 in-person learning days. Although vaccination remains the leading public health recommendation to protect against COVID-19 for those aged ≥5 years, schools might consider TTS as an option for allowing close contacts who are not fully vaccinated to remain in the classroom as an alternative to home quarantine.

**Summary**

**What is already known about this topic?**

COVID-19 transmission within K–12 schools can remain low with implementation of multiple, concurrent prevention strategies.

**What is added by this report?**

During fall 2021, 90 Lake County, Illinois, schools implemented Test to Stay (TTS), permitting eligible close contacts with masked COVID-19 exposures to remain in school. Secondary transmission among TTS participants was 1.5%; no tertiary transmission was observed among school-based contacts; however, tertiary cases were identified among household contacts. Implementation of TTS preserved up to 8,152 in-person learning days.

**What are the implications for public health practice?**

Although vaccination remains the leading recommendation to protect against COVID-19, TTS allows close contacts to remain in the classroom as an alternative to home quarantine.

## Footnotes

* Schools were provided flexibility to recommend that close contacts get off-campus nucleic acid amplification testing or antigen testing through pharmacies, clinics, or community-based testing sites during extended holidays when an interruption in the testing cadence was anticipated or if the school experienced a shortage of testing resources. In addition, some schools might have contracted private laboratories to assist in their testing program.

† Contacts with exposure to COVID-19 outside of school in the 14 days before the school-based exposure were not eligible to participate in TTS. Staff member close contacts were defined as those within 6 ft of a patient with COVID-19 for ≥15 cumulative minutes over a 24-hour period. Before October 18, 2021, a close contact on school transportation was defined by IDPH as being within 6 ft for ≥15 minutes over a 24-hour period. After October 18, 2021, the definition changed to <3 ft for ≥15 minutes over a 24 hour-period and those within 3–6 ft were no longer considered close contacts if both the confirmed patient and the close contact were masked and windows were opened or HEPA air filter was used.

§ 45 C.F.R. part 46, 21 C.F.R. part 56; 42 U.S.C. Sect. 241(d); 5 U.S.C. Sect. 552a; 44 U.S.C. Sect. 3501 et seq.

¶ Exposure locations are mutually exclusive. School nurses reported one exposure site per close contact.
